# Multifunctional metal-polymer nanoagglomerates from single-pass aerosol self-assembly

**DOI:** 10.1038/srep31329

**Published:** 2016-08-10

**Authors:** Jeong Hoon Byeon

**Affiliations:** 1 School of Mechanical Engineering, Yeungnam University, Gyeongsan 38541, Republic of Korea

## Abstract

In this study, gold (Au)-iron (Fe) nanoagglomerates were capped by a polymer mixture (PM) consisting of poly(lactide-*co*-glycolic acid), protamine sulfate, and poly-l-lysine via floating self-assembly in a single-pass aerosol configuration as multibiofunctional nanoplatforms. The Au-Fe nanoagglomerates were directly injected into PM droplets (PM dissolved in dichloromethane) in a collison atomizer and subsequently heat-treated to liberate the solvent from the droplets, resulting in the formation of PM-capped Au-Fe nanoagglomerates. Measured *in vitro*, the cytotoxicities of the nanoagglomerates (>98.5% cell viability) showed no significant differences compared with PM particles alone (>98.8%), thus implying that the nanoagglomerates are suitable for further testing of biofunctionalities. Measurements of gene delivery performance revealed that the incorporation of the Au-Fe nanoagglomerates enhanced the gene delivery performance (3.2 × 10^6^ RLU mg^−1^) of the PM particles alone (2.1 × 10^6^ RLU mg^−1^), which may have been caused by the PM structural change from a spherical to a hairy structure (i.e., the change followed the agglomerated backbone). Combining the X-ray-absorbing ability of Au and the magnetic property of Fe led to magnetic resonance (MR)-computed tomography (CT) contrast ability in a phantom; and the signal intensities [which reached 64 s^−1^ *T*_2_-relaxation in MR and 194 Hounsfield units (HUs) in CT at 6.0 mg mL^−1^] depended on particle concentration (0.5–6.0 mg mL^−1^).

Finely synthesized and modified nanoparticles in the size range of 20–500 nm are widely employed for therapeutic, diagnostic, and theranostic (i.e., both therapeutic and diagnostic) purposes. The large active surfaces of nanoparticles can improve their ability to bind and deliver functional components such as drugs, proteins, and probe materials in order to efficiently treat and diagnose cells and tissues^1^,^2^. Although numerous approaches to building such nanoparticles have been suggested, further study is still required to improve such materials’ properties and effectiveness, including preparation strategies for their practical usage.

Researchers have favored poly(lactic-*co*-glycolic acid) (PLGA) as a biocompatible platform in recent years due to its favorable physicochemical properties of stability and safety, including scalability for high production yield^3^,^4^,^5^. More recently, researchers have become interested in incorporating or combining such polymers with other functional components in order to enhance or complement their properties. Polymer-encapsulated metallic nanoparticles, in particular, are frequently employed in therapeutic and diagnostic applications^6^,^7^. Specifically, various metallic nanoparticles have been incorporated with biofunctional organic components in recent years for chemo- or photo-thermal therapies because of the former’s surface plasmon resonance heating when they are placed in cells under near-infrared (NIR) irradiation. Because NIR has high transmission efficiency in water or hemoglobin, it may be suitable for penetrating deep tissues to treat or kill cancer cells using non-invasive methods. The high thermal energies from the irradiation are capable of treating or killing cancer cells due to the thermoresponsive drug release and hyperthermic effect^7^.

Although researchers have proposed many wet chemistry formulations based on the suspension of metallic particles for biomedical applications, such formulations may only be workable (and with the desired performance) for a short period of time. In addition, organic or polymeric components incorporated with metallic nanoparticles are normally unstable due to gradual degradation by hydrolysis; biofunctional nanomaterials in a suspension or colloidal form are therefore not recommended^8^,^9^. Nanomaterials in a colloidal form also tend to aggregate during storage, thus changing the materials’ properties and making them less suitable for bioapplications^10^,^11^. As a result, we need a paradigm shift in our strategy for preparing stable organic-inorganic hybrid nanomaterials with simpler and more versatile processing for their efficient use in biomedical applications. A single-pass aerosol route is one alternative method for preparing such nanomaterials with fewer preparation steps; such a process could allow for long-term storage of the prepared nanomaterials in a powder form^12^.

Aerosol techniques in biomedical applications are generally employed for the treatment of bronchopulmonary disease via the delivery of therapeutic agents to the mucosa of the respiratory tract and pulmonary alveoli^13^; more recently, researchers have further applied and/or modified these techniques to prepare micro- or nano-scale biofunctional hybrid materials for therapeutic (e.g., drug/gene delivery) and diagnostic [e.g., magnetic resonance (MR) imaging] applications^14^,^15^,^16^. But because the conventional aerosol route of most materials is commonly performed under high-temperature conditions (at 500 °C and above), this process would only be workable for fabricating hard or inorganic materials^17^. When using a single-pass aerosol route for the production of the inorganic part of the hybrid materials, it is also essential to perform further treatments (i.e., incorporation with organic or polymeric compounds) before the materials will be suitable for use^12^. It is thus necessary to use lower-temperature processing, because temperatures above 300 °C will decompose most organic or polymeric materials (i.e., biofunctional soft materials)^18^.

This study introduces a single-pass floating self-assembly (Fig. 1) to fabricate polymer mixture (PM, consisting of PLGA, protamine sulfate, and poly-l-lysine)-capped gold (Au)-iron (Fe) nanoagglomerates as multibiofunctional nanoplatforms. In one recent study, PM nanoparticles were prepared and size-classified in the aerosol state to enhance gene delivery performance into mammalian cells without the use of multiple wet chemical processes^19^. Furthermore, the PLGA based mixture was suitable to coat along with the metal agglomerates’ surface by comparison with other aerosol polymeric particles (e.g., chitosan and its modified formulations)^20^ that prevented deep entrenching the agglomerates inside a spherical polymeric particle. More recently, Au-Fe based nanomaterials have been designed and synthesized to create multimodal theranostic nanoplatforms for biomedical applications using multiple wet chemical processes^21^,^22^,^23^,^24^. Despite the successful use of the Au and Fe (or Fe_3_O_4_, Fe_2_O_3_) components, the preparation systems of hybrid nanoplatforms should be modified with additional functional coatings and reconstructed when the compartment replacement of the platforms is required to produce a different function. Consequently, the introduction of practical generalizable assembly strategies in an on-demand configuration is a huge challenge in the preparation of various desired nanoplatforms without significant changes to the preparation system. In another study, in order to fabricate PM-based multibiofunctional nanoplatforms, Au-Fe nanoagglomerates were directly injected into PM droplets (PM dissolved in dichloromethane) in a collison atomizer and subsequently heat-treated (<90 °C wall temperature) to liberate the solvent from the droplets, thus resulting in the formation of PM-capped Au-Fe nanoagglomerates (refer to Supplementary Information); Au and Fe nanoparticles were chosen for that study since they have been highlighted as a nonproinflammatory agent^25^, and they are often employed as delivery nanocarriers or irradiation sensors for therapeutic applications^26^,^27^,^28^. Fe nanoparticles also have several potential biomedical applications, such as in MR imaging, drug targeting, and cell transfection^29^. In the current study, rapidly quenched gas-phase spark ablation operated at ambient pressure provided versatility in the assembly of inorganic-organic nanoplatforms consisting of Au-Fe nanoagglomerates and polymer mixtures at fully low-temperature conditions. Even though many reports regarding Au-Fe nanoparticles had introduced for biomedical applications, it is still highly desirable to conceive of simple, continuous, and efficient physicochemical methodologies since realizable particle modifications and on-demand preparations are urgently required for fabricating suitable biofunctional nanoplatforms for a highly profitable niche in the future nanomedicine field. The assembled PM-capped Au-Fe nanoagglomerates were electrostatically collected on hydrophobic substrates in order to evaluate their ability in gene delivery and MR-computed tomography (CT) dual-mode imaging after testing cytotoxicity.

## Results and Discussion

The particle size distribution in the aerosol-state was analyzed using a scanning mobility particle sizer (3936, TSI, Shoreview, MN, USA) to verify the mean diameter, standard deviation (SD), and number concentration (Fig. 2). The measured diameter, SD, and concentration of the PM-capped Au particles were 107.3 nm, 1.69, and 2.30 × 10^7^ cm^−3^, respectively. The other PM-capped configurations are presented in Supplementary Table S1. The data for the Au particles were 21.4 nm, 1.46, and 1.94 × 10^7^ cm^−3^, respectively, and for individual PM particles were 105.2 nm, 1.77, and 2.06 × 10^7^ cm^−3^, respectively. The similar size of PM-capped Au particles with the PM particles may be due to quantitative incorporation of Au particles with PM droplets near an orifice in the collison atomizer. The rapid changes in pressure, density, and velocity across the orifice may produce an impulse capable of redistribution of Au particles in PM matrix, resulting in the size change^30^. The results showed only a new uni-mode size distribution; the distributions were closer among the PM particles than among the Au particles, which suggests that the number concentration of PM particles was sufficient to capsulate all the Au particles for the self-assembly. The dynamic light scattering (DLS, Nano ZS90, Malvern Instruments, Worcestershire, UK) measurements of the PM-capped particles were additionally performed. The directly gas-phase sampled particles on a glass plate were applied just before biofunctionality measurements. The results showed that the deviation of hydrodynamic diameter is no larger than 5.3% for all the tested particles, and there are no significant differences between the storage days (1–14 days). This implies that the particles have stability that warrants further investigation.

In comparison with the data from the Joint Committee on Powder Diffraction Standards (not shown), the characteristic bands for Au particles at 38.2, 44.4, 64.6, and 77.5 matched well with the diffraction signals from the (111), (200), (220), and (311) of the Au crystalline facets, respectively. In the case of the Fe particles, the characteristic bands at 43.5 and 50.6 corresponded to diffraction signals from the (111) and (200) of the γ-Fe crystalline facets, respectively, while the bands at 44.4 and 64.8 were attributable to signals from the (110) and (200) of the α-Fe facets, respectively. The Au peaks overlapped with the Fe peaks in the case of the Au-Fe particles (Supplementary Fig. S1), which indicates that a simple binary metal mixture had been formed instead of an alloy^31^. The lattice parameter of Au-Fe particles obtained by the Rietveld refinement was 0.39765 nm. The results of the refinement were similar with an analogous configuration^32^ of the profile factor (*R*_p_ = 7.43%) and weighted profile factor (*R*_wp_ = 5.92%), implying that the present refinement was appropriate.

**Figure 1:**
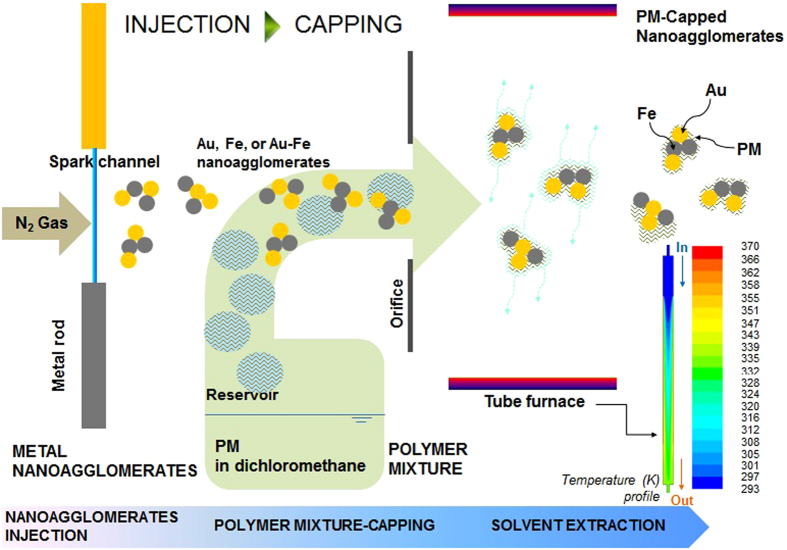
Schematic of aerosol polymer-mixture-capping of metal nanoagglomerates via floating self-assembly using a single-pass system consisting of a spark ablator and a collison atomizer.

The prepared particles (Au, Fe, Au-Fe, PM, and incorporated forms) on carbon-coated copper grids were analyzed using a transmission electron microscope (TEM) (CM-100, FEI/Philips, Hillsboro, OR, USA) in order to understand the formation of the PM-capped metal nanoagglomerates through the aerosol route. The particles were electrostatically deposited on the grid using a commercial aerosol collector (NPC-10, HCT, Icheon, South Korea). As shown in Fig. 3, the TEM observations suggest that the metal particles injected into the PM droplets in a collison atomizer were agglomerates (consisting of several primary particles), whereas the PM particles (which were well separated) exhibited a spherical shape and had a smooth surface. Metal nanoagglomerates were preferred in this study, since the agglomerates were capable of inducing a higher cellular uptake (for Au) and saturation magnetization (for Fe and Au-Fe) than those of individual nanocrystals with the same size^29^,^33^. When the metal nanoagglomerates were incorporated with the PM (around the orifice of the atomizer; refer to Fig. 1), the nanoagglomerates tended to accumulate in a PM particle, which may have been due to a higher interfacial tension between the nanoagglomerate and the PM than the tension between the nanoagglomerates, thus resulting in the PM capping of metal nanoaggolmerates. In the heated tubular flow reactor, the gas temperature (refer to Fig. 1) was somewhat higher than the glass transition temperatures of PLGA (50–60 °C); consequently, the particles were softened and a spherical morphology followed for the metal nanoagglomerates (i.e., no longer spherical)^34^. The PM coating along with the metal agglomerates’ surface may also be due to capillary force (*F*_*cap*_ = 2*πD*_*p*_*γ*cos *θ*, where *D*_*p*_ and *γ* are the particle diameter and surface tension, respectively) of voids between primary particles of the agglomerates. The conjugate points between PM and metal may also be formed via natural electrostatic charges and/or interparticle termination of propagating radicals on the metal particles during solvent extraction in the heated tubular reactor, resulting in PM morphology change^30^.

**Figure 2:**
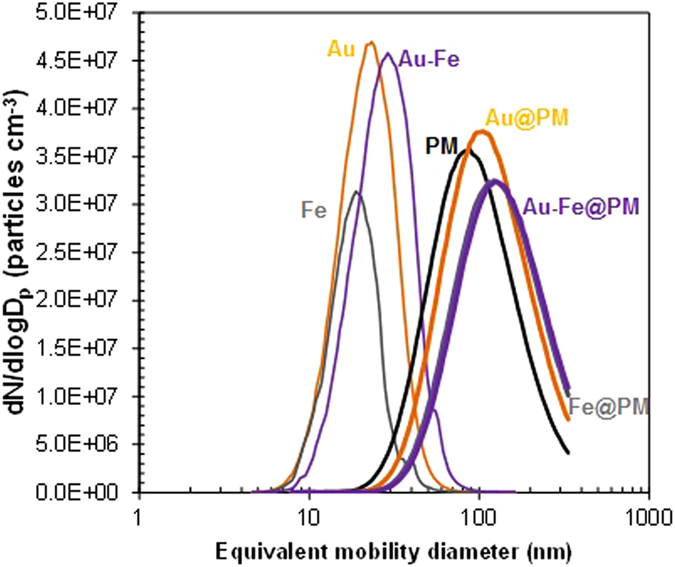
Size distributions of PM alone, metal nanoagglomerates (Au, Fe, or Au-Fe), and PM-capped metal nanoagglomerates (Au@, Fe@, or Au-Fe@PM).

**Figure 3:**
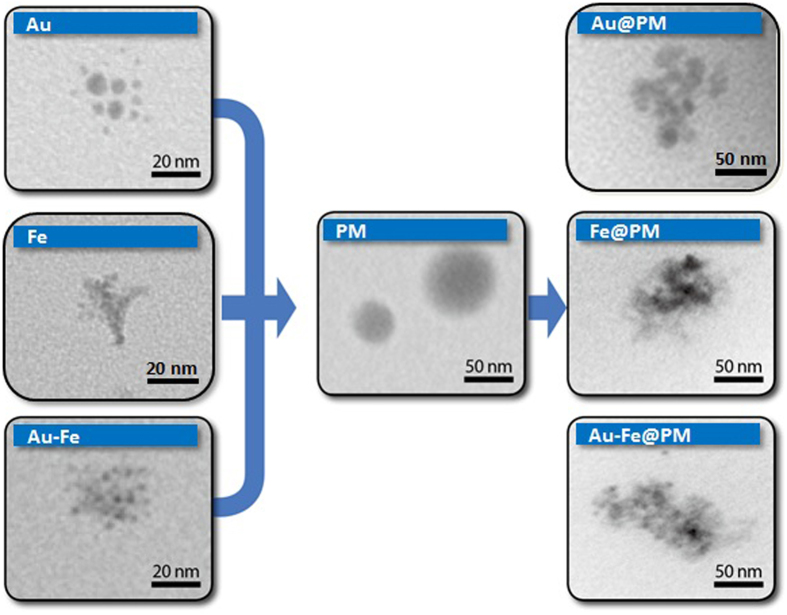
TEM images of metal nanoagglomerates (Au, Fe, and Au-Fe; **left**), PM alone (**center**), and PM-capped metal nanoagglomerates (Au@, Fe@, and Au-Fe@PM; **right**).

The average lateral dimension of the PM particles was 109 ± 5.2 nm. The analogous data for the Au@, Fe@, and Au-Fe@PM samples were 113 ± 6.2, 134 ± 8.2, and 124 ± 7.6 nm, respectively, which was comparable to the measured sizes of the samples in the aerosol state, as shown in Fig. 2. The numbers (*n*) of the PM-capped metal nanoagglomerates that were produced depended on their sizes when the sampled masses were identical; the Au@PM sample thus had the highest number of nanoagglomerates, which can be estimated by:


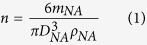


where *m*_NA_ and *D*_NA_ are the unit mass (1 mg) and the average size of the PM-capped metal nanoagglomerates, respectively, and *ρ*_NA_ is the density (approximately 1.3 g cm^−3^), which was estimated by comparison between the SMPS and experimentally weighed data using a microbalance (DV215CD, Ohaus, Greifensee, Switzerland). The mass using the SMPS can be estimated by:


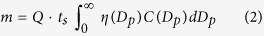


where *Q* is the flow rate of the nitrogen gas, *t*_s_ is the sampling time, *η*(*D*_p_) is the fractional collection efficiency, and *C*(*D*_p_) is the number concentration of the PM-capped metal nanoagglomerates. The estimated numbers of Au@, Fe@, and Au-Fe@PM nanoagglomerates were approximately 1.25 × 10^12^, 0.77 × 10^12^, and 0.85 × 10^12^, respectively. The surface areas of the nanoagglomerates could be further determined using this analytical approach; they were approximately 450.9, 383.4, and 396.3 m^2^ g^−1^, respectively.

As shown in Supplementary Fig. S2a, the infrared (IR) spectrum (absorbance mode) (Nicolet 6700, Thermo Electron, Madison, WI, USA) of the PM particles showed bands at around 1087 and 1187 cm^−1^, corresponding to the C-O stretch. Other bands, at 1380, 1,389, 1759, 2946, and 2960 cm^−1^, could be assigned to the C-N stretch, CH-bend, C = O ester, CH_2_-bend, and CH_3_-bend, respectively; these peaks were attributed to the PLGA backbone^35^. Protamine in the PM matrix could induce bands at around 1033 and 1190 cm^−1^, which could be indexed with symmetrical and asymmetrical stretching vibrations of S = O, respectively. Protamine with lysine in the PM matrix further introduced two more bands, at 1730 and 3400 cm^−1^, which was attributable to carbonyl ester-combined stretching and amine-hydroxyl groups, respectively^19^,^36^. The Au@PM nanoagglomerates had a similar IR spectrum (Supplementary Fig. S2b) to that of the PM alone. It was clear that the Au@PM showed relatively intense peaks at 1033 and 1190 cm^−1^ due to the greater number of S = O groups. This behavior was also shown for the Au-Fe@PM (Supplementary Fig. S2d), and may have originated from an affinity between the Au and the PM (especially the S of protamine). The Fe@PM (Supplementary Fig. S2c) showed weakened characteristic PM peaks, which also demonstrated evidence of the affinity between Au and PM.

In short, the changed spectra in the PM-capped metal samples provide evidence of the PM conjugation with the metal nanoagglomerates. The existence of the PM implies that the metal nanoagglomerates may have a DNA-binding ability to form nanoagglomerate-gene complexes via electrostatic attraction. The measured zeta potential values of the PM-capped metal-gene complexes are described in Supplementary Table S2. The positive charges of the complexes suggest that the nanoagglomarates may have potential for delivering genes into cells^37^. The zeta potential varied with capped metal species; the Au@ and Fe@PM had the highest and lowest values, respectively; these corresponded to the peak intensity of the IR spectra.

Before biofunctionality measurements, the sampled agglomerates on a glass substrate were detached in an ultrasound bath to secure stability for a long-term storage. An electrophoretic mobility shift experiment (Supplementary Fig. S3) was then performed to confirm the formation of the nanoagglomerate-gene complexes for employing such complexes in a gene delivery test. In order to investigate the appropriate ranges of mass ratio (PM-capped metal nanoagglomerate to gene), the ratios of 0.1, 0.5, 1.0, 5.0, and 10.0 were tested to confirm complexing efficiency. The 1.0 ratio (the right four lanes in Supplementary Fig. S3) was eventually chosen for the gene delivery tests, since nearly all of the genes were efficiently bound with PM-capped metal nanoagglomerates, and clearly showed little gene liberation from the complexes.

The cytotoxicity of the agglomerates was then assessed, because one essential feature of any biomedical application of PM-capped metal nanoagglomerates is that they must be biocompatible. Measurement of the cell viability of the PM-capped metal nanoagglomerates was performed at different mass concentrations (5–100 μg mL^−1^) using 3-(4,5-dimethylthiazol-2-yl)-5-(3-carboxymethoxyphenyl)-2-(4-sulfophenyl)-2H-tetrazolium (MTS) assay in 293 human embryonic kidney cells, in comparison with the cell viability from PM alone (Supplementary Fig. S4). The range of the measured cell viability was 98–103% for the nanoagglomerates (including PM alone); there were no significant differences between the nanoagglomerates and PM alone (>99% cell viability). This suggests that the incorporation of metal nanoagglomerates did not increase the cytotoxicity of the PM particles, thereby providing evidence that there was no significant interference in the cells’ reproductive abilities after incubation with the samples. The slightly higher cytotoxicities of the nanoagglomerates at higher mass concentrations might have been caused by damage via the interactions between the metal surfaces and the cellular compartments. Specifically, enzymatic degradations and/or single-strand breaks of genes in cells via catalytic oxidative stress from metal nanoagglomerates might have slightly increased the cytototoxicities at higher mass concentrations^38^,^39^. In the case of the slightly higher toxic effect in the PM-capped metal nanoagglomerates, however, it is difficult to ascertain where the toxicity arose.

**Figure 4:**
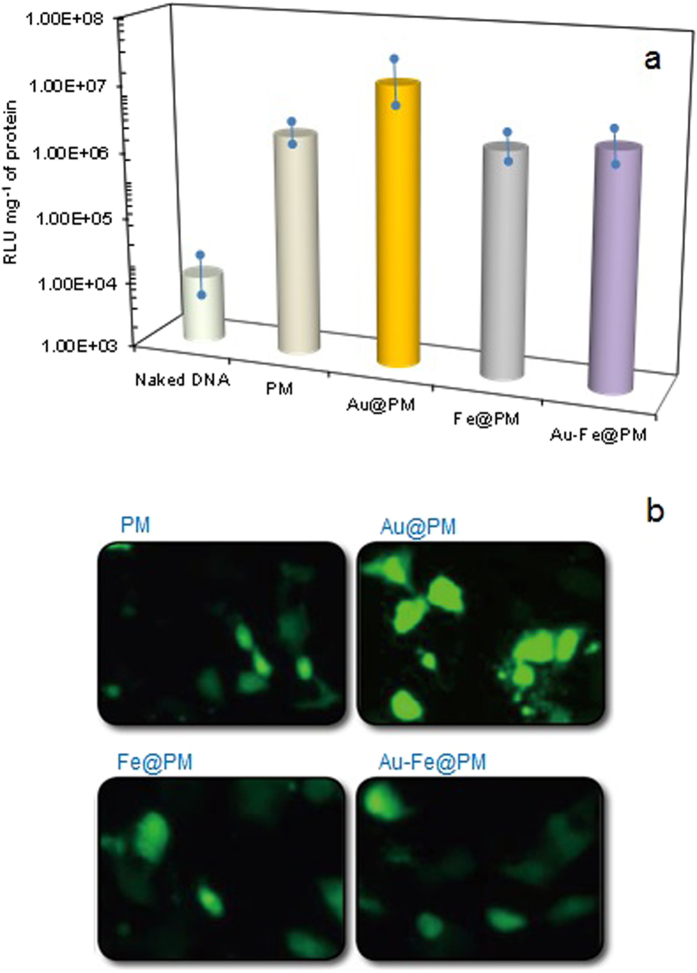
(**a**) Gene delivery performances and (**b**) the fluorescent expressions of PM alone, and PM-capped metal nanoagglomerates (Au@, Fe@, and Au-Fe@PM) in 293 human embryonic kidney cells for 24 h.

The gene delivery performance of the PM-capped nanoagglomerates was then evaluated by luminescence measurements of green fluorescent protein (GFP) genes. The results [shown in Fig. 4a, expressed as relative luminescent units (RLUs) per mg^−1^ of protein] from the PM-capped metal nanoagglomerates complexed with GFP genes were compared with the results from the PM particle-GFP gene complexes. As a control, the gene delivery performance of naked DNA was first attempted for comparison purposes; the intensity of GFP fluorescence reached approximately 1.1 × 10^4^ RLU mg^−l^ in the 293 cells. The results were significantly enhanced (i.e., intracellular transfection was achieved) by employing PM (0.21 × 10^7^ and RLU mg^−l^) and PM-capped metal nanogglomerates (>0.23 × 10^7^ RLU mg^−l^, 1.5 × 10^7^ for Au@PM; and 0.32 × 10^7^ for Au-Fe@PM); all PM-capped metal samples showed higher delivery performances. This may have been due to the PM structural change from a spherical to a hairy structure (i.e., PM-capping followed the agglomerated metal backbone), since a hairy structure can offer larger functional surfaces for more effective binding with GFP genes. As shown in Fig. 4a, the corresponding fluorescent microscope images further confirmed the different gene delivery abilities between the complexes. The Au@PM nanoagglomerates demonstrated the highest efficiency, because Au can more easily bind functionalizing or targeting ligands (i.e., inducing high affinity between the luciferase and complexes) than other materials^40^; Au@PM also has the largest surface area (450.9 m^2^ g^−1^), because it has the smallest lateral dimension (approximately 110 nm). DLS measurements (Supplementary Table S3) further support this hypothesis. One previous report indeed noted that the Au-agglomerate-incorporated polymer nanocomposites could deliver approximately six times the number of genes into COS-7 monkey kidney cells than unincorporated polymer particles^41^. One possible reason for this is that an agglomerate made of individual Au nanocrystals could interact with a cell surface receptor, either at the tip of the agglomerate (where a single nanocrystal interacts with the cell membrane) or at one of the edges (where more Au nanocrystals interact with the membrane) which may thus affect the amount and rate of nanocomposite internalization^33^. In addition, DLS was further employed to measure colloidal stability of PM-capped nanoagglomerates including pure Au-Fe in phosphate buffer solution (pH 7.4). The size of uncapped Au-Fe nanoagglomerates reached approximately 600 nm within 4 h, whereas PM-capped nanoagglomerates were remarkably stable up to 2.5 days (smaller than 200 nm) probably due to their positive (i.e., unipolar) potentials (Supplementary Table S2).

Supplementary Fig. S5 shows the magnetic properties of the Fe@PM and Au-Fe@PM nanoagglomerate samples measured by a vibrating sample magnetometer at room temperature, which indicates that the samples showed soft ferromagnetic behavior after their formation as PM-capped metal nanoagglomerates. The ratio between Au (0.415) and Fe (0.585) of the nanoagglomerates was measured using inductively coupled plasma optical emission spectroscopy (Optima 8300, PerkinElmer, USA) and energy-dispersive X-ray spectroscopy (EX-350, Horiba, Japan). The samples had saturation magnetization values (*M*_s_) of approximately 26.1 and 14.9 electromagnetic units per gram of iron (emu g^−1^) to have coercivities of 49 and 40 Oe of Fe@PM and Au-Fe@PM, respectively, and these values were smaller than the uncapped Au-Fe nanoagglomerates (86.4 emu g^−1^, implying dependence on their composition). The measured *M*_s_ of the Au-Fe@PM agglomerates was lower than the observation for Fe@PM, because Au is a diamagnetic material; therefore interparticle coupling between Fe and Au likely decreases the agglomerates’ magnetic properties^42^. The lower magnetization of Au-Fe@PM agglomerates might also be explained by an antiferromagnetic ordered core along with the presence of uncompensated surface spins for partially alloyed Au-Fe^43^.

**Figure 5:**
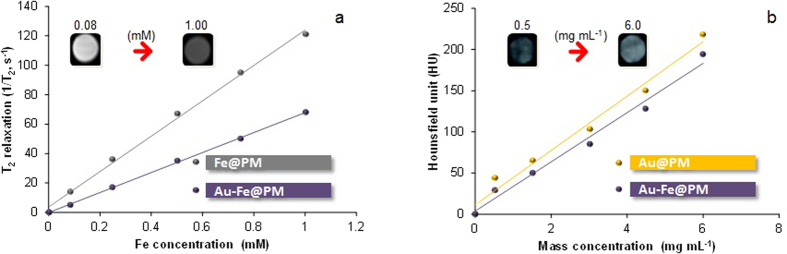
MRI and CT contrast ability of PM-capped metal nanoagglomerates: (**a**) MRI of phantoms (inset) and relaxivity plot as a function of mass concentration; (**b**) CT of phantoms (inset) and plot of X-ray attenuation in Hounsfield units (HUs) as a function of mass concentration.

After the magnetic property measurements were conducted, the dual-mode imaging performance of Au-Fe@PM nanoagglomerates was evaluated using standard MR and CT imaging instruments. MR and CT images of a phantom were measured at various concentrations (0.08–1.00 Fe mM for MR, 0.5–6.0 mg mL^−1^ for CT) of Au-Fe@PM nanoagglomerates compared with Fe@PM or Au@PM nanoagglomerates. The MR results showed increasing negative contrast in phantoms with increased Fe concentration (inset of Fig. 5a) and demonstrated a transverse relaxivity of 55.2 mMFe^−1^ s^−1^, which was smaller (corresponds to magnetic property, Supplementary Fig. S5) than the value of the Fe@PM (89.6 mMFe^−1^ s^−1^) nanoagglomerates. In the CT imaging (inset, Fig. 5b) in the phantom, the results showed increasing positive contrast when the nanoagglomerates were presented. Using X-ray absorption measurements, we found 194 HU for the Au-Fe@PM concentration of 6 mg mL^−1^, which was also not far from the value of the Au@PM (218 HU) nanoagglomerates. Since the PM-capped metal nanoagglomerates were sensitive both in MR and CT imaging, the nanoagglomerates are expected to have potential for applications in MR-CT dual-mode imaging.

This study has discussed aerosol-route fabrication of PM-capped Au-Fe nanoagglomerates via floating self-assembly of nanoscale Au-Fe and PM components used as multibiofunctional nanoplatforms. Fully low-temperature single-pass system offers flexibility concerning the combination of the resulting nanoplatforms by simply selecting appropriate electrodes and biofunctional PMs. Measurements of the nanoagglomerate’s properties demonstrated that the proposed processing facilitates multifunctionality for gene delivery and MR-CT dual-mode imaging without significant increases in cytotoxicity and size or the need for multiple chemical processes and toxic reagents. In contrast to chemical routes, the proposed system also avoids using any multiple purifications and separations, thereby allowing single-pass assembly of multicomponent biofunctional nanoplatforms with high purity in a predictable and green manner. This strategy could thus allow new on-demand fabrication and modification of fresh multifunctional nanomaterials for use in various biomedical applications, and may also hold immense promise for biomaterial coatings.

## Additional Information

**How to cite this article**: Byeon, J. H. Multifunctional metal-polymer nanoagglomerates from single-pass aerosol self-assembly. *Sci. Rep.* **6**, 31329; doi: 10.1038/srep31329 (2016).

## Supplementary Material

Supplementary Information

## References

[b1] Nel A. E. . Understanding biophysicochemical interactions at the nano-bio interface. Nat. Mater. 8, 543–557 (2009). 19525947 10.1038/nmat2442

[b2] Karmi A. . Multifunctional nanovehicles for combined 5-fluorouracil and gold nanoparticles based on the nanoprecipitation method. J. Nanosci. Nanotechnol. 11, 4675–4683 (2011). 21770092 10.1166/jnn.2011.4156

[b3] Mishra D., Kang H. C. & Bae Y. H. Reconstitutable charged polymeric micelle for gene therapeutics delivery. Biomaterials 32, 3845–3854 (2011). 21354616 10.1016/j.biomaterials.2011.01.077 PMC3073820

[b4] Blum J. S. & Saltzman W. M. High loading efficiency and tunable release of plasmid DNA encapsulated in submicron particles fabricated from PLGA conjugated with poly-l-lysine. J. Control. Release 129, 66–72 (2008). 18511145 10.1016/j.jconrel.2008.04.002 PMC2494593

[b5] Almería B. . Controlling the morphology of electrospray-generated PLGA microparticles for drug delivery. J. Colloid Interface Sci. 343, 125–133 (2010). 20022337 10.1016/j.jcis.2009.10.002

[b6] Bennewitz M. F. . Biocompatible and pH-sensitive PLGA encapsulated MnO nanocrystals for molecular and cellular MRI. ACS Nano 5, 3438–3446 (2011). 21495676 10.1021/nn1019779 PMC3102302

[b7] Furlan M. . Preparation of biocompatible magnetite-PLGA composite nanoparticles using supercritical fluid extraction of emulsions. J. Supercrit. Fluids 54, 348–356 (2010).

[b8] Craig D. Q. M. The mechanisms of drug release from solid dispersions in water-soluble polymers. Int. J. Pharm. 231, 131–144 (2002). 11755266 10.1016/s0378-5173(01)00891-2

[b9] Bulmus V. . Synthesis and characterization of degradable p(HEMA) microgels: use of acid-labile crosslinkers. Macromol. Biosci. 7, 446–455 (2007). 17429806 10.1002/mabi.200600258

[b10] Flores-Fernández G. M., Solá R. J. & Griebenow K. The relation between moisture-induced aggregation and structural changes in lyophilized insulin. J. Pharm. Pharmacol. 61, 1555–1561 (2009). 19903382 10.1211/jpp/61.11.0016

[b11] Thompson C. J. . Preparation and evaluation of microspheres prepared from novel polyester-ibuprofen conjugates blended with non-conjugated ibuprofen. J. Microencapsul. 26, 676–683 (2009). 19888876 10.3109/02652040802656333

[b12] Boissiere C. . Aerosol route to functional nanostructured inorganic and hybrid porous materials. Adv. Mater. 23, 599–623 (2011). 20963791 10.1002/adma.201001410

[b13] Edwards D. A. & Dunbar C. Bioengineering of therapeutic aerosols. Annu. Rev. Biomed. Eng. 4, 93–107 (2002). 12117752 10.1146/annurev.bioeng.4.100101.132311

[b14] Ruiz-Hernández E. . Aerosol-assisted synthesis of magnetic mesoporous silica spheres for drug targeting. Chem. Mater. 19, 3455–3463 (2007).

[b15] Colilla M. . Advanced drug delivery vectors with tailored surface properties made of mesoporous binary oxides submicronic spheres. Chem. Mater. 22, 1821–1830 (2010).

[b16] Liu J. . Electrostatically mediated liposome fusion and lipid exchange with a nanoparticle-supported bilayer for control of surface charge, drug containment, and delivery. J. Am. Chem. Soc. 131, 7567–7569 (2009). 19445508 10.1021/ja902039y PMC2724844

[b17] Iskandar F., Mikrajuddin & Okuyama K. Controllability of pore size and porosity on self-organized porous silica particles. Nano Lett. 2, 389–392 (2002).

[b18] Sanchez C. . Designed hybrid organic-inorganic nanocomposites from functional nanobuilding blocks. Chem. Mater. 13, 3061–3083 (2001).

[b19] Byeon J. H., Kim H.-K. & Roberts J. T. Monodisperse poly(lactide-co-glycolic acid)-based nanocarriers for gene transfection. Macromol. Rapid Commun. 33, 1840–1844 (2012). 22829341 10.1002/marc.201200369

[b20] Byeon J. H. . Aerosol-based fabrication of modified chitosans and their application for gene transfection. ACS Appl. Mater. Interfaces 6, 4597–4602 (2014). 24628606 10.1021/am501069u

[b21] Wang G. . Au nanocage functionalized with ultra-small Fe_3_O_4_ nanoparticles for targeting *T*_1_-*T*_2_ dual MRI and CT imaging of tumor. Sci. Rep. 6, 28258 (2016). 27312564 10.1038/srep28258 PMC4911575

[b22] Amendola V. . Magneto-plasmonic Au-Fe alloy nanoparticles designed for multimodal SERS-MRI-CT imaging. Small 10, 2476–2486 (2014). 24619736 10.1002/smll.201303372

[b23] Zhao H. Y. . Synthesis and application of strawberry-like Fe_3_O_4_-Au nanoparticles as CT-MR dual-modality contrast agents in accurate detection of the progressive liver disease. Biomaterials 51, 194–207 (2015). 25771010 10.1016/j.biomaterials.2015.02.019

[b24] Huang J. . Rational design and synthesis of *γ*Fe_2_O_3_@Au magnetic gold nanoflowers for efficient cancer theranostics. Adv. Mater. 27, 5049–5056 (2015). 26198387 10.1002/adma.201501942

[b25] Lehmann A. D. . Fluorescent-magnetic hybrid nanoparticles induce a dose-dependent increase in proinflammatory response in lung cells *in vitro* correlated with intracellular localization. Small 6, 753–762 (2010). 20205203 10.1002/smll.200901770

[b26] Li D. . Hierarchical gold/copolymer nanostructures as hydrophobic nanotanks for drug encapsulation. J. Mater. Chem. 20, 7782–7787 (2010).

[b27] Higashi N., Takagi T. & Koga T. Layer-by-layer fabrication of well-packed gold nanoparticle assemblies guided by a β-sheet peptide network. Polym. J. 42, 95–99 (2010).

[b28] Park H. . Multifunctional nanoparticles for combined doxorubicin and photothermal treatments. ACS Nano 3, 2919–2926 (2009). 19772302 10.1021/nn900215k

[b29] Jafari T., Simchi A. & Khakpash N. Synthesis and cytotoxicity assessment of superparamagnetic iron-gold core-shell nanoparticles coated with polyglycerol. J. Colloid Interface Sci. 345, 64–71 (2010). 20153479 10.1016/j.jcis.2010.01.038

[b30] Byeon J. H. & Roberts J. T. Aerosol-based fabrication of biocompatible organic-inorganic nanocomposites. ACS Appl. Mater. Interfaces 4, 2693–2698 (2012). 22509789 10.1021/am300337c

[b31] Byeon J. H., Park J. H. & Hwang J. Spark generation of monometallic and bimetallic aerosol nanoparticles. J. Aerosol Sci. 39, 888–896 (2008).

[b32] Luo Z. . Pawley and Rietveld refinements using electron diffraction from *L*1_2_-type intermetallic Au_3_Fe_1-*x*_ nanocrystals during their *in-situ* order-disorder transition. Ultramicroscopy 111, 1295–1304 (2011). 21864770 10.1016/j.ultramic.2011.04.003

[b33] Albanese A. & Chan W. C. W. Effect of gold nanoparticle aggregation on cell uptake and toxicity. ACS Nano 5, 5478–5489 (2011). 21692495 10.1021/nn2007496

[b34] Chu C.-H. . Surface deformation of gold nanorod-loaded poly(DL-lactide-co-glycolide) nanoparticles after near infrared irradiation: an active and controllable. J. Mater. Chem. 20, 3260–3264 (2010).

[b35] Zhang Y., Sun X. & Jia N. Direct electrochemistry and electrocatalysis of hemoglobin immobilized into poly (lactic-co-glycolic acid)/room temperature ionic liquid composite film. Sens. Actuator B-Chem. 157, 527–532 (2011).

[b36] Szabó T. . Layer-by-layer construction of ultrathin hybrid films with proteins and clay minerals. J. Phys. Chem. C 111, 12730–12740 (2007).

[b37] Tahara K. . Hybrid-modified poly(d,l-lactide-co-glycolide) nanospheres for a novel cellular drug delivery system. Int. J. Pharm. 392, 311–313 (2010). 20347023 10.1016/j.ijpharm.2010.03.042

[b38] Guildford A. L. . Nanoparticles of a different source induce different patterns of activation in key biochemical and cellular components of the host response. J. R. Soc. Interface 6, 1213–1221 (2009). 19324665 10.1098/rsif.2009.0021 PMC2817157

[b39] Pumera M. Nanotoxicology: the molecular science point of view. Chem. Asian J. 6, 340–348 (2011). 20725923 10.1002/asia.201000398

[b40] Lewinski N., Colvin V. & Drezek R. Cytotoxicity of nanoparticles. Small 4, 26–49 (2008). 18165959 10.1002/smll.200700595

[b41] Thomas M. & Klibanov A. M. Conjugation to gold nanoparticles enhances polyethylenimine’s transfer of plasmid DNA into mammalian cells. Proc. Natl. Acad. Sci. USA 100, 9138–9143 (2003). 12886020 10.1073/pnas.1233634100 PMC170885

[b42] Kayal S. & Ramanujan R. V. Anti-cancer drug loaded iron-gold core-shell nanoparticles (Fe@Au) for magnetic drug targeting. J. Nanosci. Nanotechnol. 10, 5527–5539 (2010). 21133071 10.1166/jnn.2010.2461

[b43] Morales M. A. . Surface anisotropy and magnetic freezing of MnO nanoparticles. Phys. Rev. B 75, 134423 (2007).

