# Supplementary material for: Multifunctional metal-polymer nanoagglomerates from single-pass aerosol self-assembly
Source: Sci Rep. 2016 Aug 10;6:31329. doi: 10.1038/srep31329 (PMC4979091; doi:10.1038/srep31329)
Supplement: Supplementary Information [file srep31329-s1.pdf]

Supplementary Information for Publication

Multifunctional metal-polymer nanoagglomerates from single-pass aerosol self-assembly

Jeong Hoon Byeon[*]

School of Mechanical Engineering, Yeungnam University, Gyeongsan 38541, Republic of Korea

METHODS

- *Nanocomposite Fabrication*

Schematics of the reactors used for these experiments are shown in **Fig. 1**. The obtained nanohybrids is composed of a metal nanoagglomerate core and an PM shell. Briefly, a spark ablation produces aerosol metal nanoagglomerates (Au, Fe, or Au-Fe). Spark ablation has been used to produce a variety of metallic, carbonaceous, and other composite materials with nanoscale dimensions at ambient temperatures and pressures.[S1-S5] The rods, which were 3 mm in diameter and 100 mm in length, were obtained from, Nilaco, Japan. The nitrogen flow rate, which was controlled by a mass flow controller (Tylan, USA), was 3 L min^{-1}. The specifications of the discharge configuration were as follows: resistance, 0.5 MΩ; capacitance, 1.0 nF; loading current 1.2 mA; applied voltage 2.6 kV; and frequency 380 Hz. A spark-produced metal nanoagglomerate-laden nitrogen (>99.99% purity) flow was used as the operating gas for atomizing a solution containing 0.2 g of PLGA (Sigma-Aldrich, USA) and 0.02 g of Prot (Sigma-Aldrich, USA) dissolved in 99.8 mL of dichloromethane (DCM, Sigma-Aldrich, USA) with 0.2 mL of PLL [0.1 % (*w*/*v*) in H_2O, Sigma-Aldrich, USA]. The metal nanoagglomerates passed over the atomizer orifice, where they mixed with atomized PM droplets to form hybrid droplets.[S6] The droplets then passed through a heated tubular flow reactor operating at a 90°C wall temperature to drive DCM from the droplets. The condition for complete evaporation can be estimated by considering the time required for the evaporation of the droplets and comparing it with the appropriate residence time in a tubular flow reactor. The characteristic time to saturate gas with vapor from evaporating droplets, τ, is given *via* the equation,[S7]

$$\tau = \frac{1}{2\pi D_d \delta_v C(D_d)} \tag{1}$$

where $C(D_d)$ is the droplet number concentration. The D_d value can be estimated *via* the equation,

$$D_d = \left(\frac{\rho_s w + \rho_p (1 - w)}{\rho_s w} D_p^3 \right)^{1/3} \tag{2}$$

where ρ_s and ρ_p are the densities of the solvent and the solid particle, respectively, and w is the weight

fraction of solid in liquid.

- *Instrumentation*

The size distributions of the aerosol particles were measured using a SMPS, consisting of an electrostatic classifier (3085, TSI, USA), ultrafine condensation particle counter (3776, TSI, USA), and aerosol charge neutralizer (4530, HCT, Korea). The SMPS system, which measures the mobility equivalent diameter, was operated at a sample flow of 0.3 L min^{-1}, a sheath flow of 1.0 L min^{-1}, and a scan time of 135 sec (measurement range: 7.91-333.8 nm). TEM images were obtained at an accelerating voltage of 46-180 kV. Specimens were prepared for examination in the TEM by direct electrostatic aerosol sampling at a sampling flow of 0.5 L min^{-1} and an operating voltage of 5 kV using a Nano Particle Collector (NPC-10, HCT, Korea). For IR analysis, samples were prepared using polytetrafluoroethylene media substrate (0.2 μm pore size, 47 mm diameter, 11807-47-N, Sartorius, Germany) by physical filtration (*i.e.* mechanical filtration mainly by diffusion, of particles on the surfaces of the substrate). The spectra were taken for samples in the range of 4000-400 cm^{-1} in absorbance mode. Samples were also characterized by measurements of magnetization by a Lakeshore 7404 vibrating sample magnetometer (VSM, USA) at room temperature (298 K). The zeta potential of sample-gene complexes was determined using a zeta potential analyzer (Nano ZS90, Malvern Instruments, UK). The PM-capped metal nanoagglomerates were mixed with gene, and incubated at room temperature for 30 min. The complexes were then diluted with double de-ionized water to an appropriate concentration. Measurements of the zeta potential were carried out at 25°C and calculated using the manufacturer's supplied software.

- *Agarose Gel Retardation Assay*

The gene condensation ability of the PM-capped metal nanoagglomerates under different weight ratios were analyzed by 1% agarose gel electrophoresis using tris-acetate-ethylenediaminetetraacetic acid buffer (242 g Tris, 57.1 mL glacial acetic acid, and 0.5 mM ethylenediaminetetraacetic acid, pH 8.0) containing

0.5 μg mL^{-1} ethidium bromide. Complexes containing PM-capped metal agglomerates and genes with different weight ratios were prepared by mixing, voltexing, and incubating them at room temperature for 30 min. Approximately 100 ng of each complex was loaded on agarose gels. A gel loading dye blue (New England BioLabs, USA) was added to each well and agarose gel electrophoresis was carried out at a constant voltage of 80 V for 50 min. The gene bands of the resultant gels were then visualized under a ultraviolet transilluminator at a wavelength of 365 nm.

- *In vitro computed tomography (CT) and magnetic resonance imaging (MRI)*

Aqueous dispersions of PM-capped metal nanoagglomerates with different mass concentrations were examined with a 4.7 T small animal MRI scanner (Bruker) to evaluate the contrast enhancement effect. T_2-weighted imaging was performed using an inversion recovery gradient echo sequence with TE = 8 ms, a slice thickness of 0.5 mm, an field of view of 3 × 3 cm, and a matrix size of 128 × 128.

CT scans were performed using GE Light Speed VCT imaging system (GE Medical Systems) operated at 100 kV and 80 mA, with a slice thickness of 0.625 mm. Dispersions with different mass concentrations were prepared in 2.0 mL Eppendorf tubes and placed in a self-designed scanning holder. Contrast enhancement was determined in Hounsfield units for each sample.

- *In Vitro Cytotoxicity and Transfection*

The cytotoxicity of the PM-capped metal nanoagglomerates was evaluated using HEK 293 cells by the MTS assay. The cells were cultured in 200 mL of Dulbecco's modified eagle medium (DMEM) supplemented with 10% fetal bovine serum (FBS) at 37℃, 5% CO_2, and 95% relative humidity. The cells were seeded in a 96-well microtiter plate (Nunc, Germany) at densities of 1×10^5 cells $well^{-1}$. After 24 h, the culture media were replaced with serum-supplemented culture media containing the PM-capped metal nanoagglomerates (1mg mL^{-1}), and the cells were incubated for 24 h. Then, 30 μL of the MTS reagent was added to each well. The cells were incubated for an additional 2 h. The absorbance was then measured using a microplate reader (Spectra Plus, TECAN, Switzerland) at a wavelength of 490 nm. The

cell viability (%) was compared with that of the untreated control cell in media without PM-capped metal nanoagglomerates and calculated with $[A]_{test}$:$[A]_{control}$ × 100%, where $[A]_{test}$ is the absorbance of the wells with PM-capped metal nanoagglomerates and $[A]_{control}$ is the absorbance of the control wells.

The ability of PM-capped metal nanoagglomerates to transfect HEK 293 cells using pDNA that contain the firefly luciferase and GFP gene. The cells were seeded at a density of 1×10^6 cells $well^{-1}$ in 24-well plate in RMPI 1640 medium (Gifco, USA) containing 10% FBS, and grown to reach 80% confluence prior to transfection. Before transfection, the medium was exchanged with fresh medium with 10% FBS. The cells were treated with the nanoagglomerates solution containing 2 μg of pDNA for 4 h at 37oC and the final volume was adjusted to 500 μL by medium. After exchanging with a fresh medium with 10% FBS, cells were further incubated for 48 h. Then the growth medium was removed, and the cells were shaken for 30 min at room temperature in 200 μL of Reporter Lysis Buffer (Promega, USA). The lysates were transferred into tubes and centrifuged at 13,000 rpm for 5 min. Luciferase activity was measured with a luminometer (TD-20/20, Promega, USA). The total protein was determined by BCA protein assay kit (ThermoFisher Scientific, USA). The final luciferase activity was expressed as RLU mg-1 protein. Inverted fluorescent microscope (Nikon Eclipse TE2000-S, Japan) was used to observe the GFP expression of the nanoagglomerates in 293 cells.

All experiments were performed in triplicate, and the results were reported as means and standard deviations. Statistical analyses were performed using Student's *t*-test. The differences were considered significant for $p < 0.05$.

Figure S1

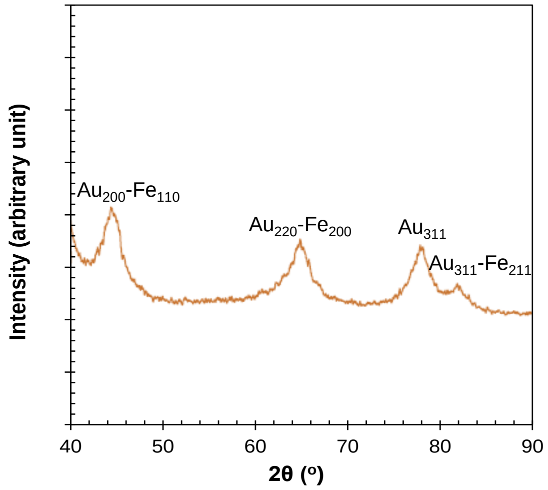

X-ray powder diffraction (D/MAX-2500, Rigaku, Japan) pattern of Au-Fe nanoagglomerates.

Figure S2

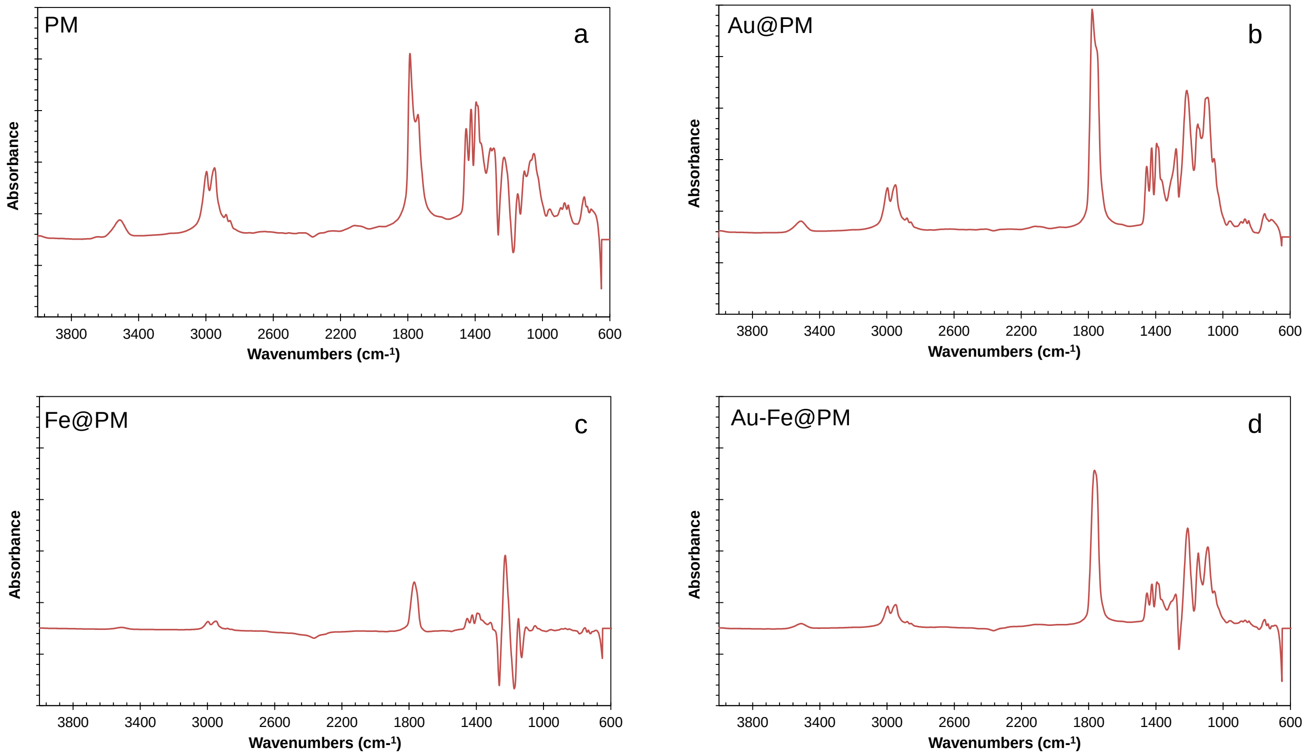

FTIR spectra of (a) PM alone, and PM-capped (b) Au, (c) Fe, and (d) Au-Fe nanoagglomerates (Au@, Fe@, and Au-Fe@PM).

Figure S3

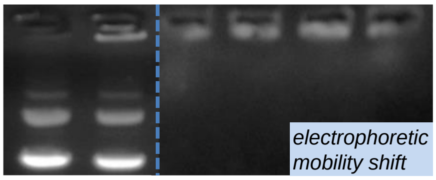

Gel retardation assay of PM-capped Au-Fe/pDNA complexes. Lane 1 is pDNA, and lanes 2-6 are PM-capped Au-Fe/pDNA complexes with the weight complex ratios of 0.1, 0.5, 1.0, 5.0, and 10.0.

Figure S4

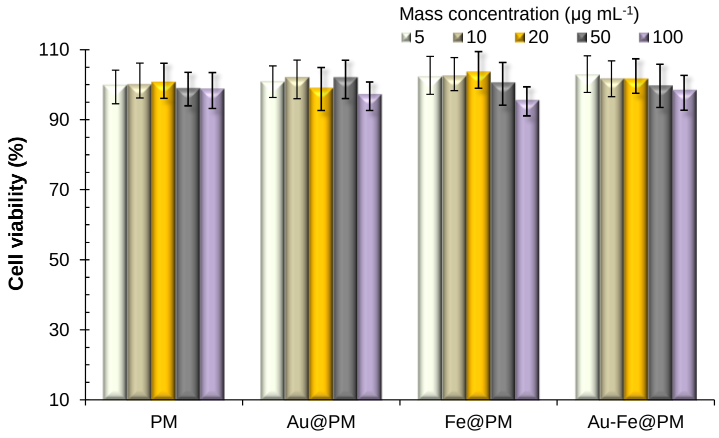

Cytotoxicity of PM alone, and PM-capped Au, Fe, and Au-Fe nanoagglomerates (Au@, Fe@, and Au-Fe@PM) in HEK 293 cells.

Figure S5

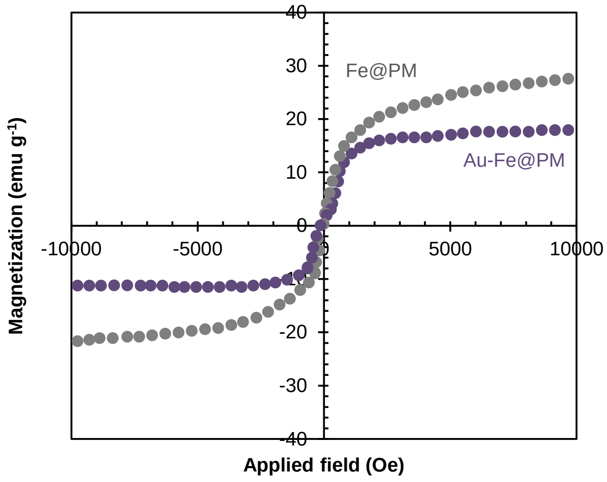

Magnetization of PM-capped Fe and Au-Fe nanoagglomerates at 293 K.

Table S1 Size distributions of PM alone, metal (Au, Fe, or Au-Fe) nanoagglomerates, and PM-capped metal nanoagglomerates (Au@, Fe@, or Au-Fe@PM)

Case	Mean diameter (nm)	Standard deviation (-)	Number concentration (cm^{-3})
PM	105.2	1.77	2.06×10^{7}
		Au@PM	
Au	21.4	1.46	1.94×10^{7}
Au@PM	107.3	1.69	2.30×10^{7}
		Fe@PM	
Fe	18.3	1.40	1.10×10^{7}
Fe@PM	126.1	1.67	1.99×10^{7}
		Au-Fe@PM	
Au-Fe	26.8	1.49	2.00×10^{7}
Au-Fe@PM	122.1	1.66	1.95×10^{7}

Table S2 Zeta potential of gene complexes with PM alone, and PM-capped metal nanoagglomerates (Au@, Fe@, and Au-Fe@PM)

Case	Zeta potential (mV)
PM	25.4 ± 3.6
Au@PM	28.3 ± 3.2
Fe@PM	14.6 ± 3.4
Au-Fe@PM	26.1 ± 3.6

Table S3 DLS measurements of gene complexes with PM alone, and PM-capped metal nanoagglomerates (Au@, Fe@, and Au-Fe@PM)

Case	Size (nm)
PM	115.2 ± 7.3
Au@PM	120.6 ± 5.5
Fe@PM	144.6 ± 6.2
Au-Fe@PM	128.9 ± 4.9

SUPPLEMENTARY REFERENCES

S1. Byeon, J. H., Park, J. H. & Hwang, J. Spark generation of monometallic and bimetallic aerosol nanoparticles. *J. Aerosol Sci.* **39**, 888-896 (2008).

S2. Byeon, J. H. *et al.* Removal of volatile organic compounds by spark generated carbon aerosol particles. *Carbon* **44**, 2106-2018 (2006).

S3. Byeon, J. H. *et al.* Ambient spark generation to synthesize carbon-encapsulated metal nanoparticles in continuous aerosol manner. *Nanoscale* **1**, 339-343 (2009).

S4. Byeon, J. H. & Kim, J.-W. Production of carbonaceous nanostructures from a silver-carbon ambient spark. *Appl. Phys. Lett.* **96**, 153102 (2010).

S5. Byeon, J. H. & Kim, J.-W. Morphology and structure of aerosol carbon-encapsulated metal nanoparticles from various ambient metal-carbon spark discharges. *ACS Appl. Mater. Interfaces* **2**, 947-951 (2010).

S6. Byeon, J. H. & Roberts, J. T. Aerosol-based fabrication of biocompatible organic-inorganic nanocomposites. *ACS Appl. Mater. Interfaces* **4**, 2693-2698 (2012).

S7. Byeon, J. H. & Roberts, J. T. Aerosol based fabrication of thiol-capped gold nanoparticles and their application for gene transfection. *Chem. Mater.* **24**, 3544-3549 (2012).
